# Atypical presentation of renal cell carcinoma with lactic acidosis: case report and literature review

**DOI:** 10.1093/jscr/rjaf290

**Published:** 2025-05-05

**Authors:** Henry Alocha, Islam Rajab, Noman Khalid, Ibraheem Sabah, Abdelfattah M Dahmas, Rawda Mahajna

**Affiliations:** Department of Internal Medicine, St. Joseph’s University Medical Center, 703 Main Street, Paterson, Passaic County, NJ 07503, United States; Department of Internal Medicine, St. Joseph’s University Medical Center, 703 Main Street, Paterson, Passaic County, NJ 07503, United States; Department of Internal Medicine, St. Joseph’s University Medical Center, 703 Main Street, Paterson, Passaic County, NJ 07503, United States; Department of Internal Medicine, St. Joseph’s University Medical Center, 703 Main Street, Paterson, Passaic County, NJ 07503, United States; Department of Medicine, Faculty of Medicine and Health Sciences, An-Najah National University, Nablus Street, New Campus, Nablus, West Bank 00970, Palestine; Department of Medicine, Faculty of Medicine and Health Sciences, An-Najah National University, Nablus Street, New Campus, Nablus, West Bank 00970, Palestine

**Keywords:** renal cell carcinoma, lactic acidosis, atypical presentation, RCC, metastasis

## Abstract

Renal cell carcinoma is often difficult to diagnose early due to its nonspecific clinical presentation, which extends beyond the classic triad of flank pain, hematuria, and a flank mass. Recognizing alternative indicators, such as microscopic hematuria and lactic acidosis, can aid in early detection. A 77-year-old male with diabetes, varicose veins, and tobacco use presented with weakness, nausea, dyspnea, and poor appetite. He exhibited somnolence, confusion, transaminitis, elevated alkaline phosphatase, and severe lactic acidosis. Urinalysis revealed microscopic hematuria. Imaging showed bilateral pleural effusions and a right hepatic lesion. A computed tomography scan identified a large renal mass invading the renal vein, inferior vena cava, and right atrium. The patient developed deep vein thromboses and underwent radical nephrectomy, but succumbed postoperatively. This case highlights renal cell carcinoma’s potential for atypical presentations, emphasizing the importance of early recognition and comprehensive diagnostic approaches to improve outcomes.

## Introduction

Renal cell carcinoma (RCC) ranks among the top 10 most common cancers in the United States and is the most prevalent form of urogenital malignancy. It predominantly affects men, with incidence rates nearly double those seen in women, and carries a significant morbidity and mortality rate, estimated to be between 30% and 40% [1, 2]. Key risk factors for RCC include obesity, hypertension, smoking, and chronic kidney disease. Despite its prevalence, RCC often escapes early detection due to its subtle and variable clinical presentations [1, 3]. This case report illustrates a rare presentation of RCC characterized by lactic acidosis and an extensive tumor thrombus. Such manifestations underscore the need for heightened clinical suspicion and comprehensive diagnostic approaches when faced with atypical clinical symptoms, thereby highlighting the complexity and deceptive nature of RCC.

## Case presentation

A 77-year-old male with a history of diabetes, varicose veins, and tobacco use presented to the emergency department with a 3-day history of generalized weakness, nausea, shortness of breath, and decreased appetite. He reported increasing lethargy and nausea over the past day, along with an 8 kg weight loss over the previous 2 months. His recent medical history included varicose vein surgery 3 weeks prior.

Upon examination, the patient was vitally stable but exhibited somnolence and confusion. The physical examination was largely unremarkable, with no dysuria or gross hematuria noted. Laboratory tests revealed transaminitis, elevated alkaline phosphatase, and significant lactic acidosis with levels between 4.8 and 6.0 mmol/L. Urinalysis showed moderate blood and more than 100 red blood cells per high-power field.

**Figure 1:**
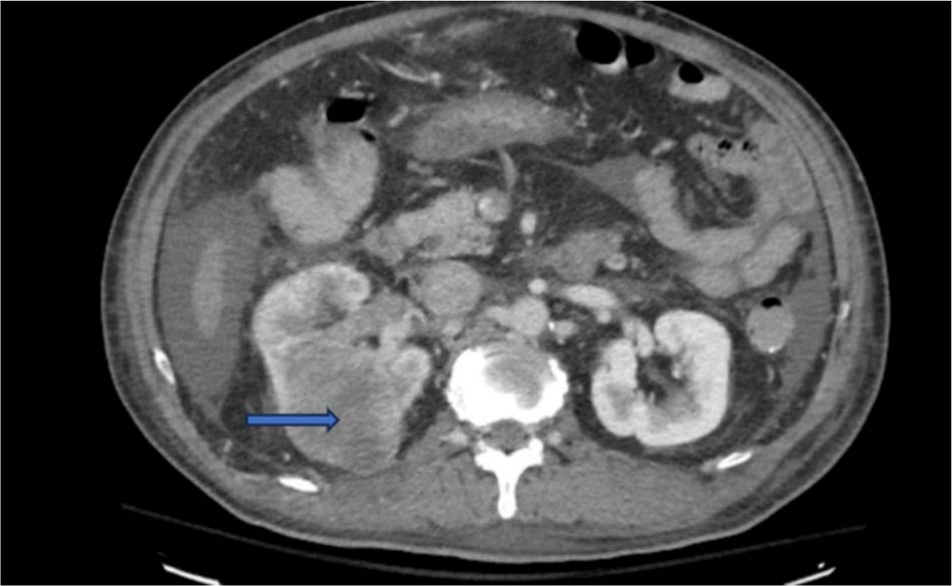
CT chest abdomen and pelvis with contrast showing middle portion of right with tumor during venous phase venous phase.

**Figure 2:**
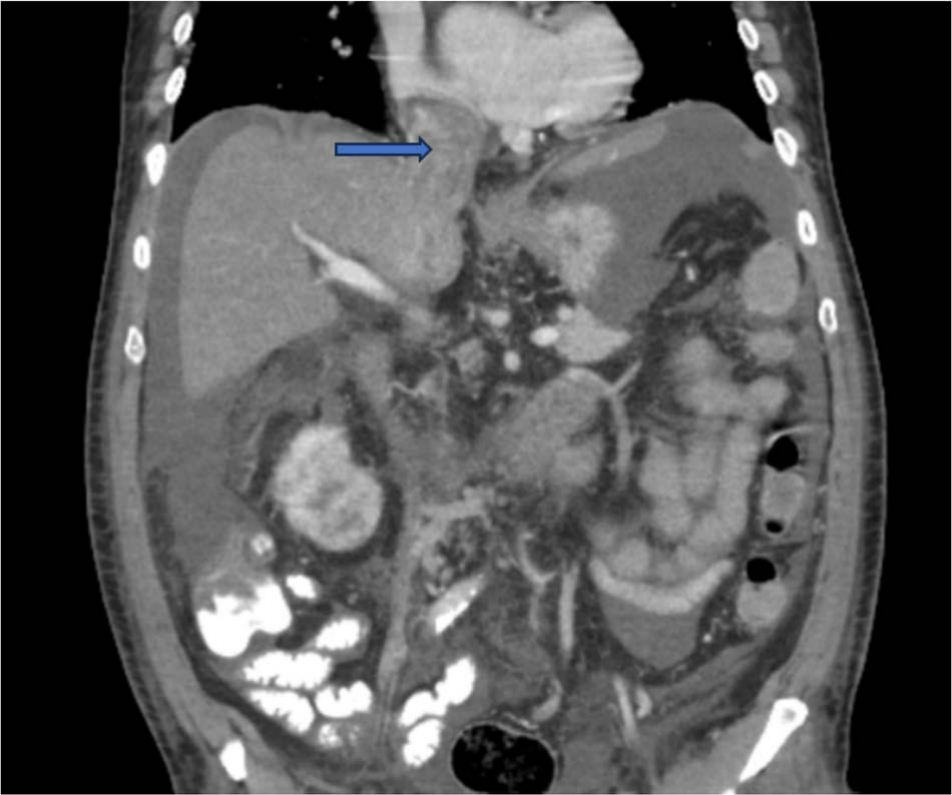
CT chest abdomen and pelvis with contrast showing tumor thrombus is seen within the IVC extending over a long distance from the level of the renal vein cephalad into the right atrium.

**Figure 3:**
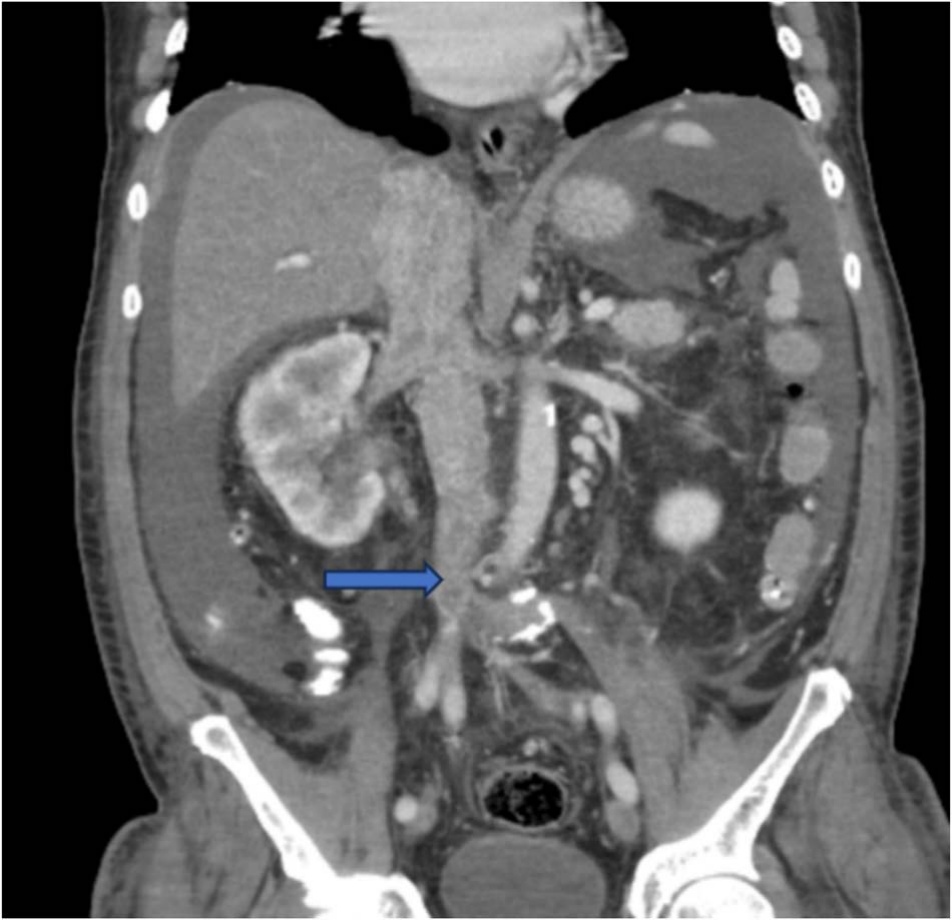
CT chest abdomen and pelvis with contrast showing inferior extension to the level of the distal margin of the IVC.

Diagnostic imaging began with a chest X-ray that indicated bilateral pleural effusions. An abdominal ultrasound detected a 4.6 × 4.2 × 4.3 cm echogenic lesion in the right hepatic lobe. Further imaging with a computed tomography (CT) scan of the chest, abdomen, and pelvis revealed a large mass originating from the posterior aspect of the right mid-kidney consistent with RCC (Fig. 1). The mass associated with thrombus extended into the renal sinus and collecting system, traveled along the right renal vein to the inferior vena cava (IVC), and projected into the right atrium (Figs 2 and 3). During hospitalization, bilateral lower extremity pain led to the diagnosis of bilateral deep vein thromboses via duplex venous ultrasound. Echocardiography showed normal left ventricular ejection fraction, impaired diastolic filling, mildly increased ventricular wall thickness, and an echogenic mass extending from the right atrium into the IVC consistent with the tumor thrombus.

For staging, a magnetic resonance imaging of the brain was performed and returned with unremarkable results. The patient was transferred to a specialized facility for radical nephrectomy and tumor thrombus removal but did not survive the procedure.

## Discussion

Hematuria, either microscopic or gross, can arise from various causes and requires careful evaluation. In patients with malignancy risk factors such as age over 35, male gender, smoking history, or exposure to carcinogenic chemicals, persistent hematuria warrants immediate investigation rather than deferred testing [4, 5]. The significant proportion of urologic cancers diagnosed through incidental hematuria underlines the importance of thorough diagnostic approaches in these cases. Our patient, exhibiting transient gross hematuria, typifies the necessity of comprehensive evaluation in high-risk individuals, a fact supported by the findings that 28.9% of older males with macroscopic hematuria have malignancies [5].

The manifestation of Type B lactic acidosis in RCC, particularly as detailed in studies by de Groot et al., underscores the metabolic impact of malignancies [6]. This type of lactic acidosis, often linked with rapid cell proliferation and insufficient metabolic compensation, presents significant diagnostic challenges [7]. The Warburg effect further elaborates on how malignancies like RCC exploit glycolysis under normoxic conditions, contributing to unusual biochemical presentations in cancer patients, include elevated serum lactate in the setting of good oxygenation (Type B lactic acidosis), and increased glucose uptake with increased lactate production due to enhanced aerobic glycolysis even in the setting of intact mitochondria [8].

Our case contributes novel insights into the complex interplay between RCC and systemic metabolic disturbances, particularly highlighting an unusual pattern of lactic acid and liver enzyme trends that began to normalize post-anticoagulation therapy. This suggests that managing thrombosis could indirectly affect tumor metabolism or local organ interactions, a finding not commonly reported in the literature. The effect of anticoagulation could be due to mild shrinkage of the extensive tumor thrombi and subsequent pressure relief from IVC on hepatic veins.

This case and literature review (Table 1) highlights the critical need for vigilance in patients presenting with atypical symptoms and known malignancy risk factors. The extensive venous involvement seen in our patient, with tumor thrombus extending into the right atrium, is a rare and serious manifestation of RCC [10]. It emphasizes the importance of comprehensive imaging and multidisciplinary approaches in the management of RCC, especially in cases involving advanced disease stages [11].

**Table 1:** Literature review of renal cell carcinoma patients presenting with lactic acidosis

**Author, year**	**Age**	**Gender**	**Type of lactic acidosis**	**Past medical history**	**Treatment**	**Follow up**
Nakajima, 2017 [7]	51	M	Type B	Unknown	Right nephrectomy, sunitinib + gemcitabine, nivolumab, everolimus	Died of complications in 5th month after everolimus initiation
Singh, 2019 [9]	56	M	Type B	Poorly differentiated metastatic neuroendocrine carcinoma, likely from the lower GI tract, RCC, GERD	Etoposide/carboplatin chemotherapy, broad spectrum antibiotics, vasopressors	Died after 16 days in ICU. Cause of death presumed to be overwhelming sepsis vs. end-organ failure from malignancies.

GERD: gastroesophageal reflux disease, GI: gastrointestinal, ICU: intensive care unit, M: male, RCC: renal cell carcinoma.

## Conclusion

The findings from our patient align with known clinical behaviors of RCC but also extend our understanding of its potential systemic effects, such as unusual presentations of lactic acidosis and the influence of venous thrombosis on tumor and organ function. This case underscores the necessity of considering RCC in differential diagnoses and tailoring patient management strategies to accommodate the breadth of possible clinical presentations and complications.
